# Paper Test Cards for Presumptive Testing of Very Low Quality Antimalarial Medications

**DOI:** 10.4269/ajtmh.14-0384

**Published:** 2015-06-03

**Authors:** Abigail A. Weaver, Marya Lieberman

**Affiliations:** Department of Chemistry and Biochemistry, University of Notre Dame, Notre Dame, Indiana

## Abstract

Carrying out chemical analysis of antimalarials to detect low-quality medications before they reach a patient is a costly venture. Here, we show that a library of chemical color tests embedded on a paper card can presumptively identify formulations corresponding to very low quality antimalarial drugs. The presence or absence of chloroquine (CQ), doxycycline (DOX), quinine, sulfadoxine, pyrimethamine, and primaquine antimalarial medications, in addition to fillers used in low-quality pharmaceuticals, are indicated by patterns of colors that are generated on the test cards. Test card sensitivity for detection of these pure components ranges from 90% to 100% with no false positives in the absence of pharmaceutical. The color intensities from reactions characteristic of CQ or DOX allowed visual detection of formulations of these medications cut with 60% or 100% filler, although samples cut with 30% filler could not be reliably detected colorimetrically. However, the addition of unexpected fillers, even in 30% quantities, or substitute pharmaceuticals, could sometimes be detected by other color reactions on the test cards. Tests are simple and inexpensive enough to be carried out in clinics, pharmacies, and ports of entry and could provide a screening method to presumptively indicate very low quality medicines throughout the supply chain.

## Introduction

### The good, the bad, and the ugly: Importance of evaluating the quality of medications.

Access to high-quality medication is part of the basic human right of access to health care.^1^ Previous studies of the quality of medications in the developing world^2^–^4^ have revealed unacceptably high prevalence of substandard and falsified drugs. Substandard drugs are here defined as drugs that do not meet pharmacopoeia standards. Falsified medicines are those that are purposefully manufactured differently than indicated by the label; in some cases, substitute pharmaceutical ingredients are present, in others, active pharmaceutical ingredients (APIs) are cut with filler such as chalk or starch. Recent reviews of published work and “grey literature” have shown that about one-third of antimalarials in sub-Saharan Africa are substandard or falsified.^3^ Many studies have found very low quality medications that are significantly underdosed, contain substitute APIs, or contain unapproved excipients.^5^–^8^ For some specific drugs, locations, and times, such as artesunate monotherapies in Laos in the early 2000s,^9^ over 80% of the products on shop shelves were found to contain little or no API. Not surprisingly, very low quality medicines have been linked to poor patient outcomes,^3^,^6^,^10^–^14^ but in a health-care setting where complications and death rates are high and resources for patient follow-up are low, these poor outcomes may only be detected when there are a lot of low-quality products in use in the same time and location. Increased vigilance in pharmaceutical screening would increase the probability of identifying such low-quality medicines before poor patient outcomes draw attention to the problem.

This manuscript describes paper test cards that could serve as an inexpensive screening tool to indicate potential low-quality antimalarial medicines in the supply chain before they reach a patient's bedside. Because there are several published methods now for field screening of artesunate antimalarials and artemisinin drugs found in artemisinin combination therapies (ACTs),^15^–^18^ we focus here on the older drugs important for clinical care of malaria in developing countries, namely chloroquine (CQ), doxycycline (DOX), quinine, sulfadoxine, pyrimethamine, and primaquine. These older antimalarials are still in use in many regions; World Health Organization (WHO) guidelines for women in areas with moderate to high malaria transmission in Africa recommend several doses of sulfadoxine/pyrimethamine during pregnancy, and seasonal malaria chemoprevention for children ages 0–5 years with sulfadoxine–pyrimethamine–amodiaquine. Drugs such as amodiaquine or lumefantrine are used as the long-lasting component in ACTs, and it is useful to have the capacity to detect their absence from such formulations. Examples of older style antimalarials with reduced and substituted ingredients have been found in studies all over the world.^2^–^4^ In addition, inexpensive antimalarials may be substituted for more expensive artemisinin derivatives in falsified ACTs.

### Paper millifluidics is a field-friendly format for running libraries of pharmaceutical quality screening tests.

Laboratory color tests are useful for qualitative analysis of many organic functional groups and inorganic species.^19^ Previous field tests for pharmaceuticals^20^,^21^ have used small-scale versions of such reactions conducted using suitcase laboratories or conventional laboratory facilities, but the equipment, chemicals, and skilled technicians needed to carry them out are often not available in low-resource settings. Here, we show that the necessary reagents and chemical manipulations needed for a dozen of these color tests can be combined on a millifluidic paper device, the size of a playing card. In a previous study, a library of 11 color tests was found to detect beta (β)-lactam antibiotics, first-line tuberculosis (TB) medications, and some pharmaceutical excipients with good sensitivity and selectivity.^22^ In this study, the test card was modified to identify aspirin (acetylsalicylic acid), which has been reported as a substitute pharmaceutical in falsified pharmaceutical formulations.^23^,^24^ Samples of pure APIs used in antimalarial drugs, formulations of APIs cut with fillers, and formulations containing substitute pharmaceuticals were applied to the test cards, and readers evaluated the blinded results to test the card's ability to identify formulations that corresponded to very low quality medications.

**Scheme 1.**
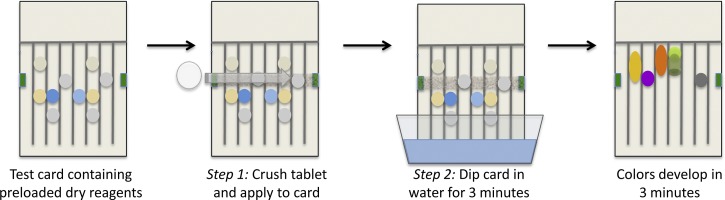
Testing a pharmaceutical with a paper analytical device (PAD). A crushed tablet or the content of a capsule is applied to the card. The card is stood upright in water, which wicks up the individual lanes to activate the color tests. After 3 minutes, the card is removed and laid flat, and a photograph is recorded in 3–5 minutes. Differences between the image of the result and images of test cards run with authentic pharmaceuticals are used to identify suspicious samples.

The test cards are used as shown in Scheme 1

**Figure 1.**
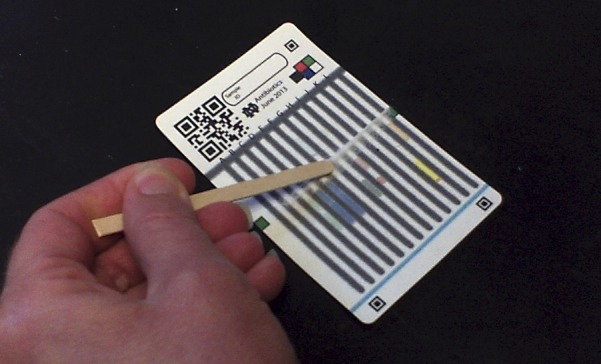
The 12-lane test card being loaded with a pharmaceutical sample.

. The porous paper provides a sturdy and light substrate, stores the needed reagents in dry form, and wicks fluids through capillary action to mix reagents and samples and chromatographically separate products. The user loads about 10 mg powder taken from a capsule or a crushed tablet by swiping it across the test card with a wooden paddle (i.e., coffee stirrer) to press small quantities of the sample into each of 12 reaction zones, as shown in Figure 1

**Figure 2.**
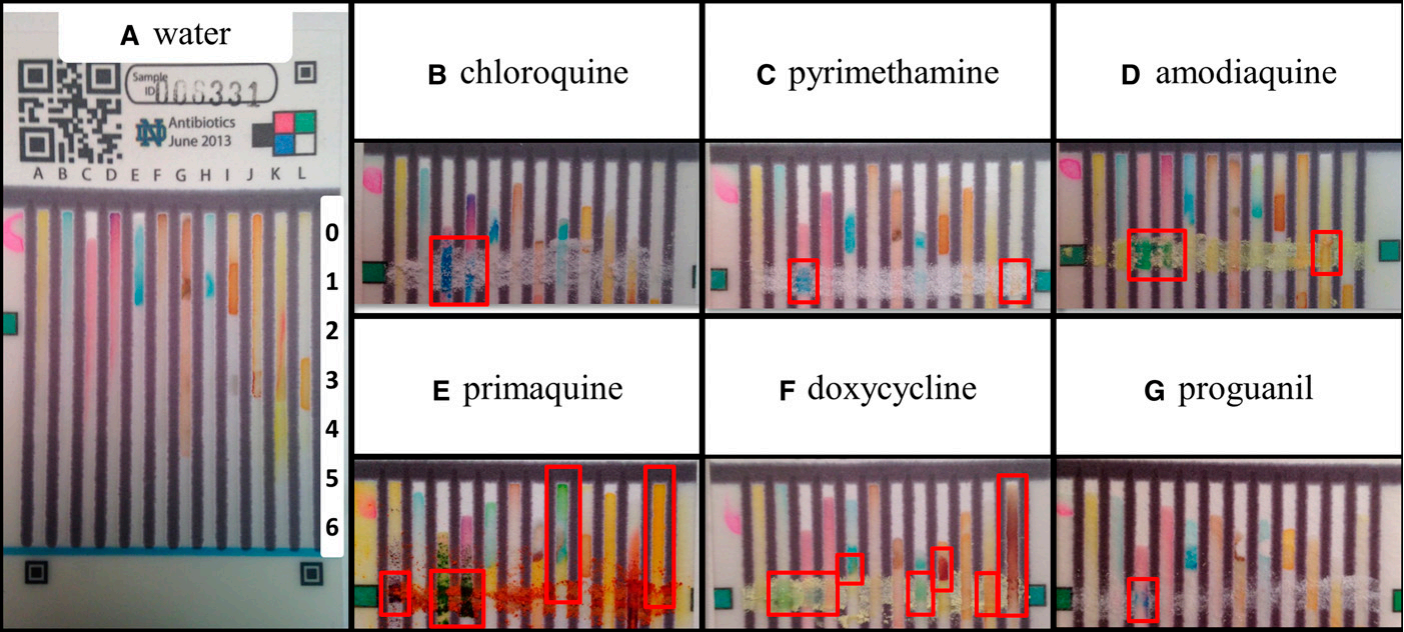
Representative outcomes of the 12-lane test card analysis of water only (**A**) and top portions of the test cards displaying the color output following testing with antimalarial standards (**B**–**G**). Outcomes of interest that differ from the water blank—the “color bar codes”—are indicated with red boxes and described in Supplemental Table 2. Images of “color bar codes” of additional pharmaceuticals and excipients can be found in Supplemental Figure 1.

. The bottom edge of the card is set into water. As the water ascends the test card via capillary action, it dissolves and combines the dried reagents stored on the card and carries them to the sample. Within 3 minutes, colors form at or above the swipe line, developing maximum intensity after another 3–5 minutes. The user records the test results by taking a photo with a cell phone camera, which documents the primary data from each test. Different mixtures of ingredients in the pill form distinct patterns of colors or “color bar codes,” as seen in Figure 2.

The question we address here is whether these color bar codes allow the user to distinguish full strength antimalarial APIs from low-quality formulations. A laboratory validation study was performed on CQ and DOX, which are non-artesunate drugs often used to treat malaria. Using a blinded study methodology, we assessed the sensitivity and specificity of the test for discrimination between authentic APIs and formulations that were diluted with fillers or contained substitute APIs.

## Materials and Methods

### Materials.

Acetylsalicylic acid, 4-pyridyl pyridinium chloride (PPC), amodiaquine, calcium carbonate, sulfadoxine, primaquine bisphosphate, pyrimethamine, atovaquone, chloroquine phosphate, quinine, polyvinylpyrrolidone (PVP) and proguanil hydrochloride were obtained from Sigma-Aldrich (St. Louis, MO). Artesunate was obtained from Tokyo Chemical Industry Co. Ltd. (Tokyo, Japan). DOX was obtained from MP Biomedical (Santa Ana, CA). Corn starch was obtained from a local grocery store. Test cards were printed on Ahlstrom 319 chromatography paper (Midlands Scientific, Chicago, IL).

The test cards were printed as described in a previous publication.^22^ A spoke inoculating manifold (or “frog” device) was obtained from Dan-Kar (Woburn, MA) and used to transfer spots of reagents stored in two 96-well plates to the printed cards.

### Methods.

The sources of chemicals, printing, and reagent deposition process used in assembling the 12-lane test card are described in previous publication,^25^ with the exception of lanes that test for aspirin and sulfadoxine and a cobalt thiocyanate test modified by the presence of a basic buffer. Unless otherwise stated, all reagents were deposited from aqueous solution. Test cards can be produced on a small scale using simple equipment, which could be suitable for local production in some countries affected by low-quality medications. Labels, instructions, fiducial marks, security features, and color standards are first printed onto the cards with a laser printer. Wax printing^26^,^27^ followed by baking in a 100°C oven is used to create hydrophobic wax barriers on the card; these barriers define reaction areas for the different tests.

Chemical reagents in 12-lane test cards were spotted in 2 μL aliquots in locations indicated in Figure 2A. The compositions and locations of the reagents are shown and described in detail in Supplemental Table 1. After air-drying the cards until the pink color of hydrated cobalt thiocyanate in lanes C and D had changed to the blue color of dehydrated cobalt thiocyanate, the cards were transferred to plastic zipper-lock bags for storage. In this study, cards were used within 4 weeks of fabrication.

Acetylsalicylic acid (aspirin) lane tests were spotted in the locations labeled in Figure 2A. The spotting reagents were: 125 mg/mL iron(III) chloride (location 1) and 6 M sodium hydroxide (location 3).

Sulfadoxine lane tests were spotted in the locations labeled in Figure 2A. The spotting reagents were: 1 M *p*-toluenesulfonic acid (locations 0, 1), 30 mg/mL PPC (location 3) and 6 M sodium hydroxide (location 4).

The compositions of samples used for the blinded study are described in Table 1. Samples were massed, combined in 20-mL scintillation vials and shaken for at least 1 minute to combine the powders.

The determination of test card results was conducted using a blinded methodology as described in previous publication.^25^ In brief, each test card was photographed 3–5 minutes after it was removed from water, and the images were masked to conceal everything except the color bar code region. Two readers compared each card to images of standard pure samples and reported whether a) the claimed API was present or absent and b) whether any adulterants were present or absent. If the two readers disagreed, a third reader was brought in as a tiebreaker. Expert readers, Abigail A. Weaver, Marya Lieberman, and Leah Koenig have interpreted > 100 test cards in a previous study.^25^ Additional readers used in this study (Margaret Berta and Murray Weaver) completed a 5- to 10-minute training. All readers compared unknown test images with standard images to determine if the outcomes are a “match” to the standard image or not. All 12-lane test cards were read by expert readers Abigail A. Weaver and Marya Lieberman with tiebreakers for active ingredient lanes read by Leah Koenig and excipient tiebreakers read by Murray Weaver. Sulfadoxine PPC lane tests were read by Abigail A. Weaver and Margaret Berta with no tiebreakers needed. The less experienced readers were used only for single-lane analysis to determine the presence/absence of a specific component. Following training, Margaret Berta and Murray Weaver interpretations from blind single-lane test reads were in 100% agreement with an expert reader.

For CQ and DOX, selected lanes were analyzed using ImageJ^28^ (NIH, Bethesda, MD) to derive color intensity data related to the content of each drug. For CQ, the mean intensity of the blue color in Lane C was analyzed in a region of interest extending 1 cm above and below the swipe line; for DOX, the mean grayscale intensity of the red-brown color of Lane L was assessed in a region of interest extending from the swipe line to the top of the lane. This analysis is facilitated by the melting behavior of the wax ink, which consists of a mixture of colorless hydrophobic waxes and pigments; on melting, the colorless waxes permeate the paper at a faster rate than the pigments, so each gray hydrophobic barrier is surrounded by a halo about 0.5-mm wide of uncolored but hydrophobic paper, which repels colored products that are chromatographically mobile in the water phase. It is thus possible to draw a boundary box around the entire colored portion of the lane without including pixels from the gray barrier.

## Results

### Versatility in detection of antimalarial APIs, substitute APIs, fillers, and excipients.

The color tests assembled on the paper test card signal the presence of APIs and excipient materials found in common antimalarial drugs, producing a color bar code that reflects the dosage form composition. In this study, test cards were found to produce unique color bar codes for CQ, pyrimethamine, DOX, quinine phosphate or quinine-free base (same color bar code), atovaquone, proguanil, amodiaquine, and primaquine standards. Different color bar codes are produced by substitute pharmaceuticals and fillers used in some previously observed falsified formulations, such as acetylsalicylic acid, acetaminophen, starch, and calcium carbonate. Representative images of test outcomes are found in Figure 2 and Supplemental Figure 1, and annotated descriptions are given in Supplemental Table 2, describing the characteristic differences in colors generated in the lanes with each material tested. To characterize the color bar codes, pure antimalarial compounds were run in triplicate on 12-lane test cards. Two readers judged how color production differed from a water blank, and the consensus was used to define a color bar code for each compound described in Supplemental Table 2. Some color bar codes are strong and distinctive, such as CQ, which produces a strong blue color that extends above and below the sample swipe line in the two cobalt thiocyanate containing test lanes (Figure 2B, 3rd and 4th lanes). Other compounds produce weaker or less distinctive color bar codes. Proguanil produces a small amount of blue color on the swipe line in the acidic cobalt thiocyanate lane (Figure 2G, 3rd lane), but this color is not distinctive enough to constitute a color bar code, because other molecules that contain tertiary amine groups also give it. Some APIs such as amodiaquine, primaquine, and DOX are themselves colored, and in these cases the sample color at the swipe line provides an additional layer of information when combined with the colors produced by chemical reactions on the paper device.

### Ability to detect formulations corresponding to very low quality medications.

A blinded study methodology was used to assess pure CQ or DOX that was combined with different amounts of fillers (Table 1 and Figure 4) to correspond to full strength (100%) and substandard (70% and 40%) formulations. Additional samples in the pool consisted of a substitute API (aspirin, acetylsalicylic acid) or an inert filler (chalk, CaCO_3_). Two expert readers, who have analyzed over 100 test card images, were independently presented with images of the test cards in random order. The readers assessed the images by comparison to sets of standard images of water, pure CQ, pure DOX, pure acetylsalicylic acid, and pure calcium carbonate. A third reader was used in 7–8% cases where readers disagreed. In the DOX study, the readers disagreed about the excipients present in 4/60 images but agreed on all 60 images about the presence/absence of DOX; in the CQ study, they disagreed about the presence of excipients in 6/80 images and agreed on all 80 images about the presence/absence of CQ. In addition, selected lanes were analyzed using ImageJ to derive color intensity data, as described in the Methods section.

**Figure 3.**
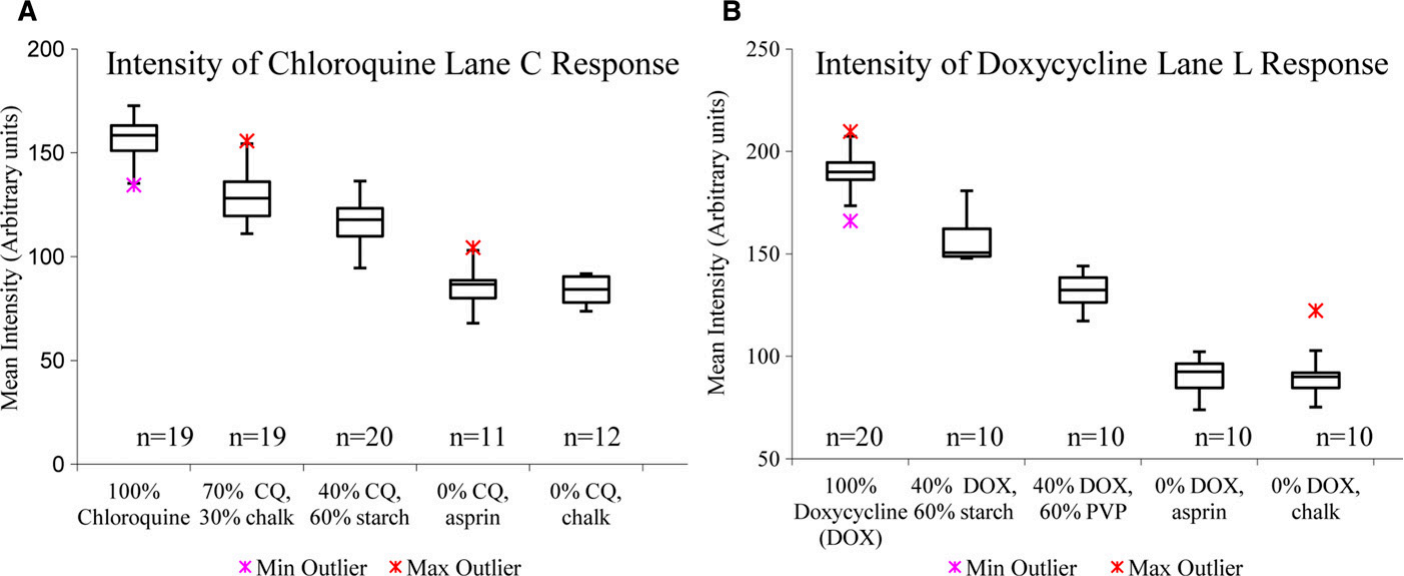
(**A**) Blue color of chloroquine (CQ) in Lane C region of interest measured as mean intensity of inverted image in red channel. (**B**) Brown color of doxycycline (DOX) in Lane L region of interest measured as mean intensity of inverted image in gray scale. Box plots depict the median (horizontal line), second and third quartile (box), and 1.5 × interquartile range (whiskers) for each sample group; sample sizes are provided below each box. Minimum and maximum outliers shown with crosses.

In assessing a test result, readers judge whether the claimed active ingredient is present or absent and whether an unapproved filler or substitute ingredient is present. The readers identified the presence of CQ by eye with 100% sensitivity (all samples containing CQ in any concentration were identified correctly, *N* = 60) and 100% specificity (no false–positive outcomes occurred for samples that did not contain CQ, *N* = 20) (Supplemental Table 3). This demonstrates the ability of the test to discriminate presence/absence of CQ with visual inspection. The mean intensities of the blue color of CQ in the images of Lane C showed significantly different values for full strength (157 ± 10, mean ± standard deviation [SD]) and 40% API (117 ± 10) formulations, as shown in Figure 3A

**Figure 4.**
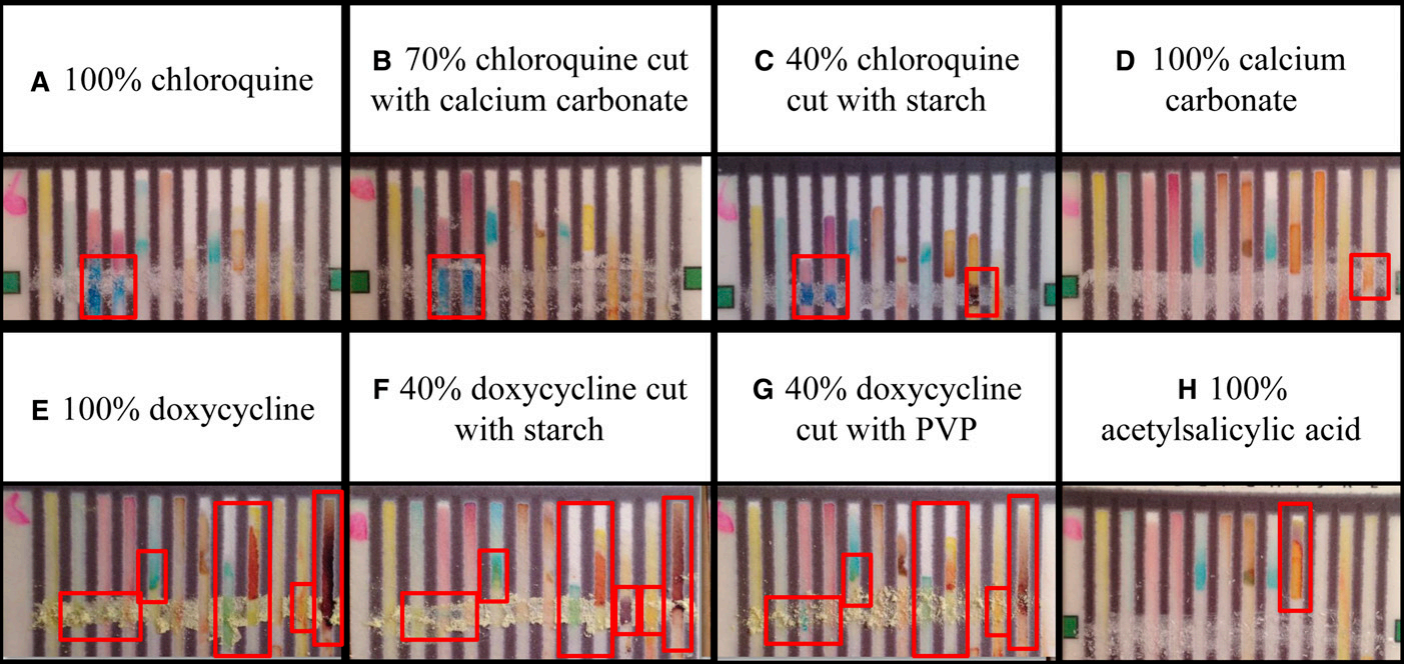
12-lane test card outcomes for samples representing different pharmaceutical formulations. Chloroquine (CQ) at 100% (**A**) looks very similar to 70% CQ/30% calcium carbonate, however 40% CQ/60% starch (**C**) produces a different pattern of colors because of the lighter blue color in the third lane and the presence of starch. 100% calcium carbonate (**D**) gives an orange color with iron chloride, but this color is not detectable in the 30% calcium carbonate used to cut CQ (**B**). The active pharmaceutical ingredient (API) doxycycline (DOX) at 100% produces a distinctive color bar code (**E**). At 40% concentration, the intensity of the brown color in the last lane is much weaker (**F** and **G**) and the appearance of starch (**F**) alters the overall color bar code and would indicate a variation in this formulation. Acetylsalicylic acid (**H**) produces a unique color bar code and is easily distinguished from antimalarial APIs.

, suggesting that readers should be able to glean semiquantitative information from test card results. An experienced reader reevaluated the blinded samples, this time rating each CQ as “strong,” “weak,” or “missing.” All of the 18 full-strength formulations were rated as “strong,” while 6/19 samples containing 70% API were rated as “strong” and 13/19 as “weak.” Of the 20 samples containing 40% API, one was rated as “strong” and the other 19/20 as “weak.” This result confirms that an experienced reader could pick out formulations containing less than 50% API by visual examination. However, the reader could not reliably distinguish full strength and 70% formulations by eye.

To aid in distinguishing dosage forms with reduced active ingredients, the 12-lane test card is equipped with several lanes that detect common binders, fillers, and substitute APIs. When an active ingredient is reduced, it is generally replaced by another compound, and this substitution may lead to a change in the color bar code. Pure calcium carbonate and pure acetylsalicylic acid, which could be added as inexpensive fillers in an adulterated dosage form, were identified from test cards by readers with 100% and 90% sensitivity, respectively, and with no false positives (Supplemental Table 3). The presence of starch in samples of 40% CQ:60% starch were all identified by eye based on the presence of a strong purple-black color produced in Lane J. Profiling both active ingredients and fillers adds specificity to the test card results. However, this profiling may be complicated in cases in which approved formulations are variable. Some CQ dosage forms include microcrystalline cellulose and talc as excipients, while others include starch as the excipient.

DOX created several characteristic color changes in different regions of interest, including pale green at the swipe line in Lane H, a bright orange color in Lane I, and a deep brown-red in Lane L (Figure 2F). Through visual inspection, readers identified formulations containing DOX with 100% sensitivity (all of the samples containing any concentration of DOX were identified correctly, *N* = 40) and specificity (no false positives from the samples lacking DOX, *N* = 20). Pure DOX could be distinguished from 40% DOX cut with corn starch (representing a very low quality drug) with 95% sensitivity and 100% specificity by evaluation of the blue-black color of starch in Lane J, but samples containing 40% DOX cut with PVP, a filler that does not produce distinct colors with the current test card, were not easily distinguished and specificity fell to 10%. However, the brown color produced by the reaction of DOX with Fe(III) in Lane L provided additional information to help distinguish 100% DOX (grayscale color intensity189 ± 10, mean ± SD) from 40% DOX (color intensity 132 ± 8) formulations (Figure 3B). An experienced reader reevaluated lane L of the DOX images, scoring the brown color in each as strong, weak, or absent. Of the 20 full-strength formulations of DOX, 19 were rated “strong” and one “weak.” The 10 formulations containing 40% DOX cut with PVP were all rated “weak,” as were 8 of the 10 formulations containing 40% DOX cut with starch (the other two were rated “strong”).

### Lane test development for sulfadoxine.

**Figure 5.**
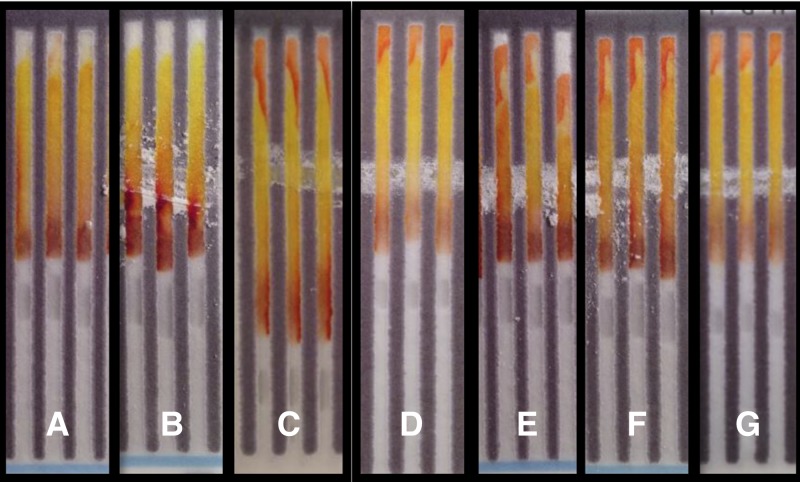
Test for primary aromatic amines produces a bright orange at the top of the test lane with 100% sulfadoxine (**F**) and a sulfadoxine/pyrimethamine combination (**G**) compared with water (**A**) or starch (**B**). Sulfadoxine cut with calcium carbonate (w/w) to concentrations of 10% (**C**), 40% (**D**), and 70% (**E**) also produce the positive outcome with only small reductions in sensitivity.

Sulfadoxine and pyrimethamine are sold in a combination dosage form. Pyrimethamine produces a strong color bar code with the test card and in its pure form could be identified with 95% sensitivity; however, the sulfadoxine/pyrimethamine combination dosage form contains only 3.5% pyrimethamine by weight. In this very dilute combination form, the test card could only identify 40% samples containing pyrimethamine (Supplemental Table 3), so it would not be reliable for identification of sulfadoxine/pyrimethamine formulations that were missing pyrimethamine. Sulfadoxine produced a very weak color bar code on the original test card (Supplemental Figure 1 and Supplemental Table 2) so an additional lane was developed to indicate the presence of the primary aromatic amine in this drug. PPC and sodium hydroxide were used to generate glutaconic aldehyde in situ just below the swipe line; the aldehyde condenses with the aromatic amine of sulfadoxine in the presence of acid to yield an intensely orange product located at the top of the test lane (Figure 5).^19^ This test is able to detect sulfadoxine with greater than 90% sensitivity at concentrations as low as 10% in a formulation cut with calcium carbonate (Supplemental Table 4).

## Discussion

There is a long history of using color reactions to characterize pharmaceutical ingredients or formulations^21^,^29^ in low-resource settings, but these methods require a high level of support infrastructure and technical skill. Here, we have translated a library of chemical color reactions onto a paper test card for presumptive testing of very low quality medications. The cards are designed to be easy to use to screen a wide variety of pharmaceuticals. The test card described here includes 13 color reactions that build a chemical profile of the major ingredients present in pharmaceutical dosage forms. Building this profile or “color bar code” increases specificity of the test by reflecting both active and inactive components present in a sample. Comparison of test card outcomes to the expected color bar code allows the user to identify differences in dosage form composition that could be potentially harmful to a patient. The library of color reactions has been shown to identify several β-lactam antibiotics and four of the first-line TB drugs,^22^ and we now show that they can identify eight non-artemisinin antimalarial drugs and three substitute APIs and excipient materials as well. More importantly, full-strength DOX or CQ could be distinguished from very low quality formulations where the API had been cut with fillers to concentrations of 40%, or replaced with substitute APIs or inert ingredients.

Disadvantages of the test cards include the need for comparison to standard outcomes of authentic samples, the existence of many pharmaceuticals that do not give useful color bar codes, the lack of sufficient quantification to identify substandard medications, and the necessity to establish a confirmatory testing process. Different brands and dose levels may use different excipients, which can alter “color bar codes,” increasing the number of potential standard outcomes the user must access for comparison with unknown samples. In addition, the user may not know the putative brand of the drug that should serve as a standard for comparison, as loose pills are sometimes dispensed without packaging information in the developing world. For these drugs, detection of excipients will be of less use than for drugs where the expected formulation is known. Some APIs that are detected readily in concentrated form do not produce strong color responses when they are used as minor constituents of a combination tablet, as seen with pyrimethamine present at 3.5% in sulfadoxine/pyrimethamine formulations. Other APIs, such as artemether, or excipients, such as gypsum, do not give color bar codes with the current set of test lanes. Finally, the test results are presumptive, and suspicious samples must be analyzed by a “gold standard” method to confirm that a drug is of poor quality.

Advantages of the test cards include ease of use and ability to build and share digital libraries of standard outcomes. Paper test cards allow presumptive testing of pharmaceuticals outside the laboratory. Users do not have to weigh or dilute anything or handle any chemicals. The test cards are inexpensive (manufacturing costs under $0.50 per card^25^) and do not require purchase of any capital equipment, such as readers or kits of glassware, so the financial barrier to use the cards is minimal. The turnaround time for analysis of a dosage form is under 10 minutes. Standards for comparison may include cards run with authentic samples, or stored images that may be shared commonly among test users.

At present, the default situation in many developing countries is that pharmaceuticals are credence goods—the buyer must take their quality on faith. Often, low-quality medications are only detected after they harm enough patients to alert medical caregivers.^12^,^14^ A widespread postmarket screening program would bring suspicious pharmaceuticals to attention and allow further regulatory and legal actions to be taken more rapidly. An initial path to implementation of such a screening program could be through governmental or nongovernmental organizations that either purchase or monitor large quantities of medications in low-resource settings. These might include ministries of health, pharmaceutical manufacturer associations, country medical regulatory authorities, the WHO, and nongovernmental organizations with health missions. These organizations have or could develop the infrastructure needed to sample and screen drugs in a field setting, have access to resources for confirmatory testing, and have capabilities for follow-up with manufacturers and regulatory agencies. The availability of more tools, such as paper test cards, for presumptive testing of pharmaceuticals could provide an inexpensive and sustainable mechanism to decrease the prevalence of low-quality pharmaceuticals in the global supply chain.

## Supplementary Material

Supplemental Datas.

## Figures and Tables

**Table 1:** Composition of active and inactive components in mixed samples

Active ingredient(s) (percent by mass)	Inactive ingredients (percent by mass)
70% CQ	30% calcium carbonate
40% CQ	60% corn starch
70% sulfadoxine, 3.5% pyrimethamine	26.5% corn starch
70% sulfadoxine	30% calcium carbonate
40% sulfadoxine	60% calcium carbonate
10% sulfadoxine	90% calcium carbonate
40% DOX	60% corn starch
40% DOX	60% PVP

CQ = chloroquine; DOX = doxycycline; PVP = polyvinylpyrrolidone.
