# Supplementary material for: Paper Test Cards for Presumptive Testing of Very Low Quality Antimalarial Medications
Source: Am J Trop Med Hyg. 2015 Jun 3;92(Suppl 6):17–23. doi: 10.4269/ajtmh.14-0384 (PMC4455083; doi:10.4269/ajtmh.14-0384)
Supplement: Supplementary file 1 [file SD1.pdf]

Supplemental Table 1
Layout of test reagents for 12-lane PAD

	Lane	Row	Reagent	Concentration (in water unless stated otherwise)
Timer	Edge	0	$NiCl_2$	0.2 M
		3	Dimethylglyoxime	0.2 M in acetonitrile
Ninhydrin	A	1	Ninhydrin	50 mg/mL in acetonitrile
		2	Ninhydrin	50 mg/mL in acetonitrile
		3	Ninhydrin	50 mg/mL in acetonitrile
		5	K_2CO_3	2 M
Biuret reagent	B	3	Biuret test reagent*	10×
		4	Biuret test reagent*	10×
Tertiary amines	C	3	$Co(SCN)_2$	1 M $Co(SCN)_2$
		4	$Co(SCN)_2$	1 M $Co(SCN)_2$
		5	Tosic acid	1 M
		6	Tosic acid	1 M
Basic $Co(SCN)_2$	D	3	$Co(SCN)_2$	1 M $Co(SCN)_2$
		4	$Co(SCN)_2$	1 M $Co(SCN)_2$
		5	Tris buffer pH 8.0	1 M
		6	Tris buffer pH 8.0	1 M
β-lactams (Cu)	E	1	$CuSO_4{\cdot}5H_2O$	1 M
		2	K_2CO_3	2 M
Sodium nitroprusside	F	1	Sodium nitroprusside	200 mg/mL
		3	Sodium nitroprusside	200 mg/mL
		4	NaOH	2 M
Naphtholquinone sulfonate	G	1	NaOH	6 M
		3	NQS	10 mg/mL
		4	NQS	10 mg/mL
Ethambutol (Cu)	H	1	NaOH	2 M
		3	$CuSO_4{\cdot}5H_2O$	1 M
Aspirin test	I	1	$FeCl_3$	125 mg/mL in water
		3	NaOH	6M
Starch	J	3	Triiodide/povidone	0.5% I_2, excess I–, 2% povidone
p-Nitroaniline	K	3	NaOH	2 M
		4	$NaNO_2$	30 mg/mL
		5	*p*-Nitroaniline	30 mg/mL in acetonitrile
		6	Tosic acid	1 M
Carbonate	L	3	$FeCl_3$	125 mg/mL in water
Sulfadoxine		0	Tosic acid	1 M
		1	Tosic acid	1 M
		3	Pyridyl pyridinium chloride	30 mg/mL
		4	NaOH	6 M

PAD = paper analytical device; SNP = sodium nitroprusside; NQS = naphthoquinone sulfonate.
See Figure 2A for locations of lanes and rows referred to in this table.
*Biuret test reagent 10× recipe: 1.5 g CuSO4·5H2O, 6 g Rochelle salt, 20 mL 2 M NaOH.

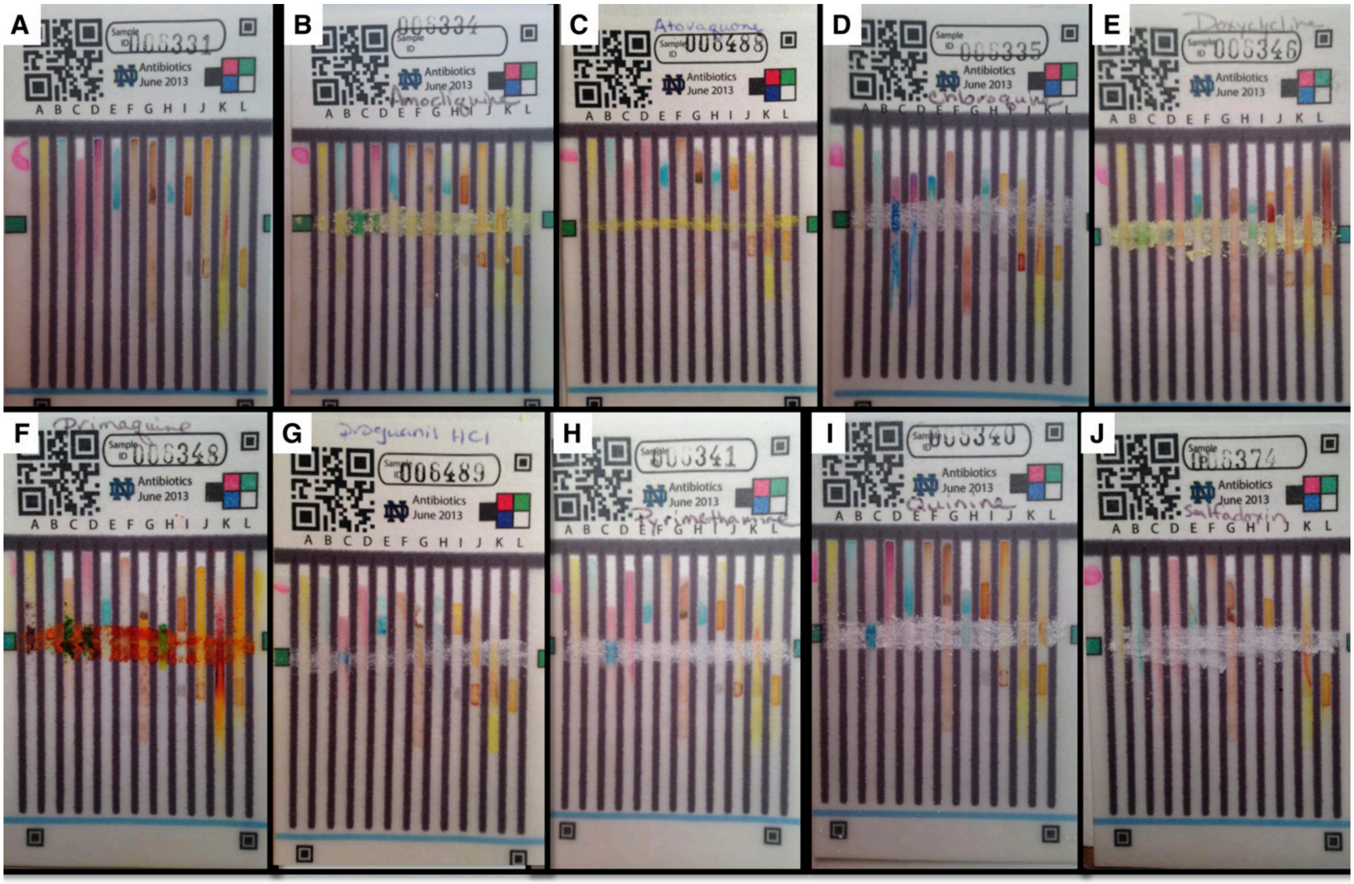

Supplemental Figure 1. Images of 12-lane paper analytical devices (PADs) run with different analytes. Analytes include water (**A**), amodiaquine (**B**), atovaquone (**C**), chloroquine (**D**), doxycycline (**E**), primaquine (**F**), proguanil (**G**), pyrimethamine (**H**), quinine (**I**), and sulfadoxine (**J**).

Supplemental Table 2

Lane by lane test outcomes for antimalarial active ingredients

Sample	Sample color	Timer (edge)	Ninhydrin (Lane A)	Biuret (Lane B)	Cobalt thiocyanate acidic (Lane C)	Cobalt thiocyanate pH 8.0 (Lane D)	β-lactams (Lane E)
Water	None	Pink spot	Yellow	Light blue	Pink	Pink	Blue (does not always reach top of lane)
Amodiaquine	Pale yellow	Like water	Like water	Like water	Emerald green at swipe	Emerald green at swipe, (fainter than Lane C)	Like water
Artesunate	White	Like water	Like water	Like water	Like water	Faint blue at test edges on swipe	Like water
Atovaquone	Bright yellow	Like water	Like water	Like water	Like water	Like water	Like water
Chloroquine	White	Like water	Like water	Like water	Blue at swipe	Blue at swipe (fainter than Lane C)	Like water
Doxycycline	Pale yellow	Like water	Like water	Green at swipe	Pale green at swipe	Faint orange at swipe	Green just above swipe
Primaquine	Bright orange	Yellow background pink spot	Black at swipe	Like water	Dark green at swipe	Dark green at swipe	Like water
Pyrimethamine	White	Like water	Like water	Like water	Blue at swipe	Like water	Like water
Proguanil	White	Like water	Like water	Like water	Blue at swipe	Like water	Like water
Quinine and quinine sulfate	White	Like water	Like water	Like water	Blue at swipe	Like water	Like water
Sulfadoxine	White	Like water	Like water	Like water	Like water	Like water	Green streak at top of blue

	SNP (Lane F)	NQS (Lane G)	Ethambutol (Lane H)	Aspirin test (Lane I)	Starch test (Lane J)	*p*-Nitroaniline (Lane K)	Iron(III) chloride (Lane L)
Water	Brown to top of lane	Dark brown spot above swipe, light brown above	Blue	Orange	Yellow/orange	Traces of orange/ yellow near and below swipe	White at swipe and above
Amodiaquine	Like water	Like water	Traces of green in blue	Like water	Like water	Yellow at swipe, faint yellow above swipe	Like water
Artesunate	Like water	Like water	Like water	Like water	Like water	Like water	Like water
Atovaquone	Like water	Like water	Like water	Like water	Like water	Orange at swipe line	Like water (compound is yellow)
Chloroquine	Like water, reduced mobility	Reduced mobility, little to no color above swipe	Like water	Like water	Like water	Like water	Like water
Doxycycline	Like water	Like water	Green at swipe	Deep orange above swipe	Like water	Orange at swipe line	Brown
Primaquine	Like water	Yellow at swipe, above is like water	Green at or above swipe	Like water	Like water	Red at swipe	Yellow
Pyrimethamine	Like water	Like water	Like water	Like water	Like water	Like water	Faint yellow a swipe
Proguanil	Like water	Like water	Like water	Like water	Like water	Like water	Like water
Quinine and quinine sulfate	Like water	Like water	Like water	Like water	Like water	Like water	Yellow at swipe
Sulfadoxine	Like water	Like water	Like water	Like water	Like water	Like water	Like water

SNP = sodium nitroprusside; NQS = naphthoquinone sulfonate.

Supplemental Table 3

Samples used and subsequent 12-lane test card sensitivity and specificity based on blinded reader evaluations. Sample numbers are shown in parentheses following sensitivity and specificity

	Positive test samples	Negative test samples	Sensitivity	Specificity	Reader disagreements
Presence of active ingredient	100%, 70%, 40% CQ	100% calcium carbonate, 100% acetylsalicylic acid	100% (60/60)	100% (20/20)	0
	100%, 70% CQ	40% or less CQ	100% (40/40)	50% (20/40)	0
	100% pyrimethamine	100% calcium carbonate, 100% acetylsalicylic acid	95% (19/20)	100% (20/20)	1
	Sulfadoxine pyrimethamine mixture (for pyrimethamine)	100% calcium carbonate, 100% acetylsalicylic acid	40% (8/20)	100% (20/20)	1
	100%, 40% DOX	100% calcium carbonate, 100% acetylsalicylic acid	100 (40/40)	100% (20/20)	0
	100% CQ	70% CQ with calcium carbonate	100% (20/20)	0% (0/20)	5
	100% DOX	40% DOX with starch	95% (19/20)	100% (10/10)	1
	100% DOX	40% DOX with PVP	95% (19/20)	10% (1/10)	3
Presence of excipients	100% acetylsalicylic acid	100%, 70%, and 40%, CQ, 100% calcium carbonate	90% (9/10)	100% (70/70)	22
	100% calcium carbonate	100%, 40% CQ, 100% acetyl salicylic acid	100% (10/10)	100% (50/50)	1
	40% CQ with starch	CQ without starch (100%, 70%)	100% (20/20)	100% (40/40)	1

CQ = chloroquine; DOX = doxycycline; PVP = polyvinylpyrrolidone.

Supplemental Table 4

Sulfadoxine test lane sensitivity and specificity based on blinded reader evaluations

	Positive test samples	Negative test samples	Sensitivity	Specificity	Reader disagreements
Presence of active ingredient	100% sulfadoxine	No API (corn starch)	100% (24)	100% (24)	0
	100%, 70% sulfadoxine	No API (corn starch)	98% (48)	100% (24)	0
	70% sulfadoxine	No API (corn starch)	96% (24)	100% (24)	0
	40% sulfadoxine	No API (corn starch)	100% (24)	100% (24)	0
	10% sulfadoxine	No API (corn starch)	92% (24)	100% (24)	0

API = active pharmaceutical ingredient.
